# Rapid and asymmetric divergence of duplicate genes in the human gene coexpression network

**DOI:** 10.1186/1471-2105-7-46

**Published:** 2006-01-27

**Authors:** Wen-Yu Chung, Reka Albert, Istvan Albert, Anton Nekrutenko, Kateryna D Makova

**Affiliations:** 1 Department of Computer Science and Engineering, Penn State University, University Park, PA, 16802, USA; 2 Department of Physics, Penn State University, University Park, PA, 16802, USA; 3 Department of Biochemistry and Molecular Biology, Penn State University, University Park, PA, 16802, USA; 4 Department of Biology, Penn State University, University Park, PA, 16802, USA; 5 Department of Huck Institute for Life Sciences, Penn State University, University Park, PA, 16802, USA; 6 Department of Center for Comparative Genomics and Bioinformatics, Penn State University, University Park, PA, 16802, USA

## Abstract

**Background:**

While gene duplication is known to be one of the most common mechanisms of genome evolution, the fates of genes after duplication are still being debated. In particular, it is presently unknown whether most duplicate genes preserve (or subdivide) the functions of the parental gene or acquire new functions. One aspect of gene function, that is the expression profile in gene coexpression network, has been largely unexplored for duplicate genes.

**Results:**

Here we build a human gene coexpression network using human tissue-specific microarray data and investigate the divergence of duplicate genes in it. The topology of this network is scale-free. Interestingly, our analysis indicates that duplicate genes rapidly lose shared coexpressed partners: after approximately 50 million years since duplication, the two duplicate genes in a pair have only slightly higher number of shared partners as compared with two random singletons. We also show that duplicate gene pairs quickly acquire new coexpressed partners: the average number of partners for a duplicate gene pair is significantly greater than that for a singleton (the latter number can be used as a proxy of the number of partners for a parental singleton gene before duplication). The divergence in gene expression between two duplicates in a pair occurs asymmetrically: one gene usually has more partners than the other one. The network is resilient to both random and degree-based *in silico *removal of either singletons or duplicate genes. In contrast, the network is especially vulnerable to the removal of highly connected genes when duplicate genes and singletons are considered together.

**Conclusion:**

Duplicate genes rapidly diverge in their expression profiles in the network and play similar role in maintaining the network robustness as compared with singletons.

*Contact: *kdm16@psu.edu

*Supplementary information: *Please see additional files.

## Background

Approximately half of human genes are members of duplicate gene families [1] and such genes might be playing an important role in the robustness of organisms against mutations (reviewed in [2-4]). Do most duplicate genes retain the functions of their parental singleton gene? Or do they diverge after duplication? And how rapidly does this divergence occur? Several models predicting preservation of both duplicate gene copies (e.g., gene function conservation, subfunctionalization, neofunctionalization, and subneofunctionalization) have been proposed [5-7] and reviewed in [8,9], however, their relative prevalence in the fates of duplicate genes is presently unknown. Originally, these questions were addressed by analysis of the protein-coding sequences of duplicate genes. Namely, the pattern of nonsynonymous *vs*. synonymous substitutions between duplicate genes was used to predict divergence in function (e.g., [9-11]). However, protein-coding sequences possess only partial information about gene evolution and function. The availability of genome-wide mRNA expression data allows one to study another important aspect of duplicate gene evolution, that is divergence in gene expression after duplication (e.g., [12-15]).

The divergence of duplicate genes in gene coexpression networks, where connectivity is based on similarity in gene expression patterns (e.g., [16-18]), represents yet another facet of duplicate gene evolution that awaits detailed investigation. Gene coexpression networks as well as many other biological networks (e.g., metabolic and protein-protein interaction networks) were shown to be scale-free [16,17,19,20]: the topology of these networks is dominated by a relatively small number of highly connected nodes, also called hubs [21,22]. Scale-free networks were found to be tolerant against random removal of nodes, but particularly vulnerable to preferential removal of hubs [23]. The studies of the evolutionary origins of scale-free biological networks suggested that gene duplication can lead to both network growth and preferential attachment and to result in a scale-free topology [22,24-26]. Thus, duplicate genes are likely to be the major players in the evolution of biological networks and investigation of their divergence in these networks is of great importance. So far, the divergence of duplicate genes has only been examined in yeast transcriptional regulation and protein-protein interaction networks [9,27-30], however, it has not been explored in networks of more complex organisms, e.g., mammals.

In mammals, where genome-wide transcriptional regulation and protein-protein interaction data are limited [31,32], coexpression networks provide an alternative for investigation of duplicate gene divergence at the systems biology level (protein-protein interaction and transcription regulatory links are a subset of links in gene coexpression networks). Coexpression and functional relationship of genes are expected to be positively correlated. Indeed, clustering of mRNA expression data has been successfully used for grouping genes similar in function [33,34] and a global correlation was found between gene expression and protein-protein interaction data [35,36]. Additionally, thousands of coexpression connections between genes were found to be evolutionarily conserved among distant organisms [18], again suggesting a strong link between similar expression pattern and functional relatedness. However, some individual links between genes might not represent direct functional relationships due to the noisiness of microarray data and network transitivity.

In the present study we build a human gene coexpression network based on human tissue-specific microarray data. We examine the divergence of duplicate genes in this network by addressing the following questions: (1) are duplicate genes or singletons represented more frequently among network hubs; (2) how rapidly do duplicate genes lose shared parental partners; (3) how quickly do they acquire new coexpressed partners; (4) is the divergence in the gene coexpression network symmetric or asymmetric between two duplicate genes in a pair; and (5) do duplicate genes and singletons play different roles in maintaining the robustness of this network.

## Results and discussion

### Description of the network

To build the gene coexpression network, we used the mRNA expression data that provide information about ~45,000 transcripts assayed in 79 human tissues [37]. We mapped probe sets to genes (see Methods) and as a result obtained a data set with one-to-one probe set to gene correspondence. This data set consisted of 14,342 genes, including 261 tissue-specific and 3460 ubiquitously expressed genes.

Two genes (represented by nodes) were connected by an edge if (1) both of them were simultaneously expressed in at least *T *common tissues, and (2) the Pearson correlation coefficient of their logarithmically transformed (with base 2) expression values was greater than or equal to *R *[16]. Nine networks were constructed depending on the combination of *T *and *R *(*T *≥ 5, *T *≥ 7, or *T *≥ 9; and *R *≥ 0.5, *R *≥ 0.7, or *R *≥ 0.9). Here in addition to the Pearson correlation coefficient we used a threshold of the minimal number of common tissues in which both genes are expressed. Relying on the correlation coefficient alone could lead to non-biological artifacts, e.g., artifactual similarities based on non-expression or expression in a few tissues only. Thus, by adding this additional criterion we obtain a meaningful correlation coefficient as it is calculated from at least five data points and only for tissues in which both genes are expressed (AD>200). To characterize the global topology of these networks, we used several graph measures [38]. *First*, the average node degree <*k*> reflected the average number of genes expressed together with a given gene. *Second*, the average shortest path length <*d*> specified the average number of edges required to travel from one gene to any other gene. *Third*, the average clustering coefficient <*c*> measured the connectivity of the neighborhood of a gene. With increases in *T *and *R*, the number of genes in the main cluster and the average number of genes coexpressed with a given gene decreased, while the average shortest path length increased (Additional file 1).

**Figure 1:**
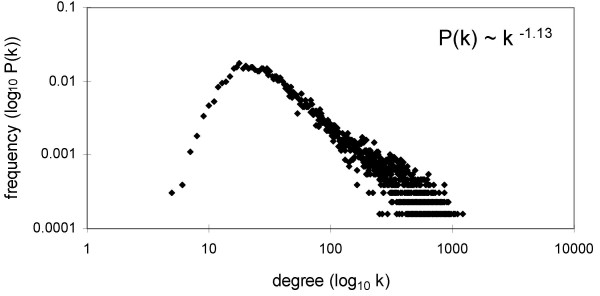
**Degree distribution of the studied network (T ≥ 7 and R ≥ 0.7).**   The degree distribution of the studied network.

Additionally, we investigated the node degree distribution *P*(*k*) describing the frequency of the number of genes with *k *coexpressed genes. For five (out of nine) networks, this distribution approximated a power law distribution (Figures 1 and Additional file 2), a characteristic of scale-free networks [21]. We used the network with *T *≥ 7 and *R *≥ 0.7 for further examination, since for these thresholds the degree distribution had a power law tail (Figure 1) and we still retained a large number of genes for a statistical analysis (however, our main conclusions hold for all thresholds examined). This network contained 12,897 nodes (all located in the main cluster) with the average degree of 132.4 (Table 1). The density (the number of observed connections divided by the number of possible connections) of the present network is 0.0103, which is comparable to the value of 0.0057 obtained for a human gene coexpression network consisting of ~9,000 genes and confirmed by at least three microarray data sets [34].

**Table 1:** Description of the studied network and the differences between duplicate genes and singletons

Gene categories	Number of nodes in the giant cluster (*n*)	Average degree (<*k*>)	Average shortest path length (<*d*>)	Average clustering coefficient (<*c*>)
All genes	12897	132.40	2.64	0.16
Duplicate genes	6507	120.11	n/a	0.14
Singletons	6390	144.92	n/a	0.17

**Figure 2:**
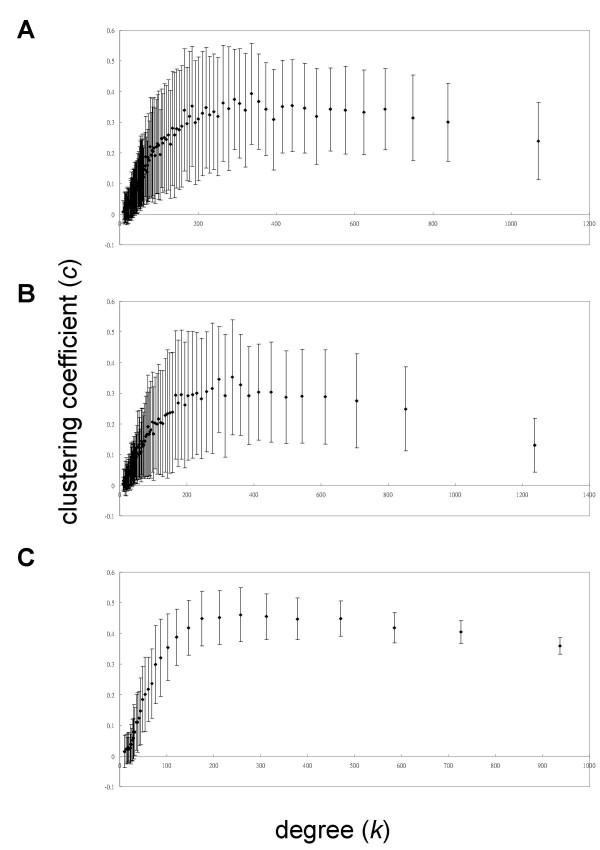
**The relationship between clustering coefficient *c *and node degree *k *for (A) all genes, (B) ubiquitously expressed genes, and (C) nonubiquitously expressed genes**. Each point represents an average value for 100 genes.

Interestingly, we found a complex relationship between clustering coefficient *c *and degree *k*: *c *increased steadily with increasing *k *for *k *< ~300, then it slowly decreased (Figure 2A and Additional file 3). The increasing relationship was more pronounced since it represented a larger sample of nodes. This implied that genes with a moderately high number of coexpressed genes usually had highly connected neighbors. This observation was unexpected as other scale-free networks display either negative or no correlation between clustering coefficient and degree [22]. Initially we suspected that the relationship observed here could be explained by a large number of ubiquitously expressed genes that have a high probability of being clustered among themselves and with other genes. However, a largely positive correlation between *c *and *k *was observed for either ubiquitously expressed or non-ubiquitously expressed genes (Figures 2B and 2C), suggesting that this is a general property of the studied network.

### Differences between duplicate genes and singletons

**Figure 3:**
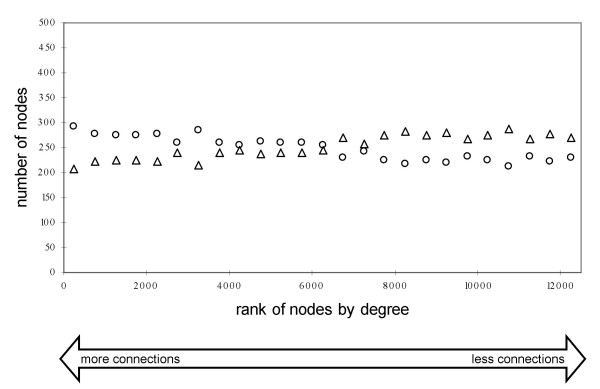
**The number of duplicate genes and singletons in every 500 genes ranked by degree**. Duplicate genes are marked by triangles and singletons are marked by circles. The genes with the highest degree are shown at the left side of the figure.

A total of 11,512 duplicate genes were identified among 22,103 Ensembl (NCBI build 34) known and novel proteins (see Methods). The studied network consisted of 6,390 singletons and 6,507 duplicate genes. Interestingly, while genes in the two categories had similar degree distributions (Additional file 4), duplicate genes had a lower average node degree and thus were less connected in the network than singletons, although the difference was small (102.1 *vs*. 144.9; Table 1; *t *= -7.74; *P *< 0.001 as assessed by permutation test). Ranking the nodes by degree indicated that among highly connected genes the proportion of singletons was higher than the proportion of duplicates (Figure 3). Thus, the effect of increased copy number might be more severe for genes with numerous coexpressed partners in the network and, as a result, duplications of such genes might have a lower propensity to become fixed in a population as compared with duplications of genes with few connections [27]. Interestingly, the average clustering coefficient was significantly lower for duplicates than for singletons (Table 1; *t *= -9.86; *P *< 0.001 as assessed by permutation test). This suggests a lower likelihood of a duplication fixation for a gene that has a tightly connected neighborhood. In this network, the percentage of duplicate gene pairs with at least one Gene Ontology [39] term overlap was higher among pairs connected by a link *vs*. unconnected pairs (97% *vs*. 86%). This is a much higher percentage than that observed for singletons – either linked (22%) or unlinked (15%). Thus, duplicate genes, especially if they are linked, had greater functional similarity.

Among the strongest hubs for duplicates, the proteins participating in nucleotide, nucleic acid, ATP, and protein binding were overrepresented (determined from the Gene Ontology terms). In addition to these categories, mitochondrion, signal transduction, and membrane proteins were overrepresented among singleton hubs. Interestingly, similarly to duplicate hubs, duplicates with the lowest number of links were involved in protein and ATP binding. However, singletons with a small number of links had different functions: e.g., receptor, transcription factor and transcription regulation activity.

### Duplicate genes rapidly lose shared coexpressed partners

**Figure 4:**
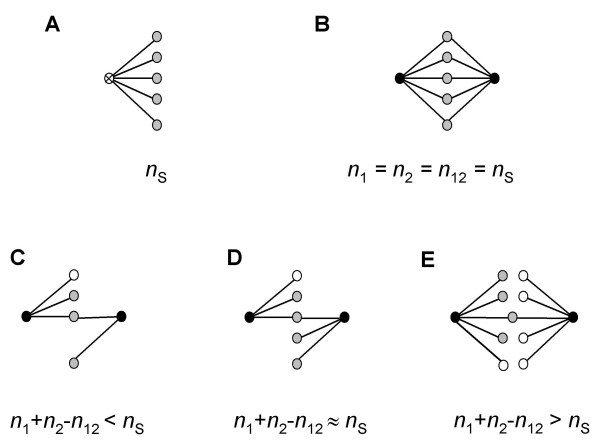
**The schematic representation of duplicate gene evolution (A) prior to duplication event, (B) immediately after duplication, (C, D, E) after some time following gene duplication**. The ancestral singleton gene is shown with a crossed line, duplicate genes are in black, shared ancestral partners are in grey, unique ancestral partners are in stripes, and unique acquired partners are in white; *n*_*s*_, *n*_1 _and *n*_2 _are the numbers of partners for a singleton, first duplicate, and second duplicate, respectively; *n*_12 _is the number of shared partners for a duplicate gene pair.

We investigated the dynamics of loss and gain of coexpressed partners (genes expressed together with a given gene, henceforth called *partners*) between two duplicate genes constituting a pair. We denoted the number of partners of one and the other duplicate gene in a pair as *n*_1 _and *n*_2_, respectively, and the number of partners shared between the two duplicate genes as *n*_12 _(Figure 4). We assumed that immediately after duplication, each duplicate gene was expressed together with *n*_1 _= *n*_2 _= *n*_12 _other genes (Figure 4B). With time, duplicate genes lose shared partners and acquire new ones. Here we assume that shared partners in a duplicate pair are inherited from a parental gene.

**Figure 5:**
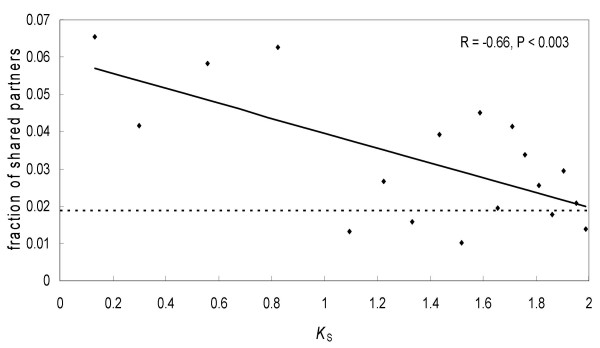
**The change in the fraction of shared partners with evolutionary time (measured by *K*_S_)**. Each point represents an average value for 40 duplicate gene pairs. Dashed line indicates the fraction of shared partners averaged among 1000 randomly selected pairs of singletons (random selection process was repeated 1000 times).

We discovered that duplicate genes lose shared partners rapidly with evolutionary time. We calculated the fraction of shared partners among all partners for each duplicate gene pair, *n*_12_/(*n*_1 _+ *n*_2 _- *n*_12_), and used the synonymous rate per site, *K*_S_, as a proxy of evolutionary time since gene duplication (Figure 5). For this analysis we used the 698 independent duplicate gene pairs (i.e. each gene was present only once in this data set, see Methods) for which both genes were present in the network and *K*_S _was less than 2. Our initial observation was that the fraction of shared partners for duplicate genes within each pair was usually low: it was <20% for 666 out of 698 duplicate pairs studied (the highest fraction of shared partners for a duplicate gene pair was 68%). A significant negative correlation was observed between *n*_12_/(*n*_1 _+ *n*_2 _- *n*_12_) and *K*_S _(*R *= -0.66, *P *< 0.003). The fraction of shared partners for a duplicate pair was on average 6.6% after only ~50 million years (MY) since duplication (this corresponds to *K*_S _= 0.13 and requires an assumption that human and Old World monkeys diverged ~25 MY ago and the sequence divergence between them is ~7%, [40]). At *K*_S _≈ 2, this fraction approached 1.9%, and the partners for the two duplicate genes in a pair were as different as those for a pair of unrelated singletons (Figure 5).

Several factors could have affected our results. At low *K*_S_, the fraction of shared partners could have been underestimated because the youngest duplicate genes were excluded from the analysis due to the lack of unique microarray probes (see Methods). At high *K*_S_, some of the shared partners could have been acquired independently (i.e. convergently) by each gene in a duplicate pair and not inherited from a parental gene. And finally, our assumption of identical expression profiles for two daughter duplicate genes immediately after duplication might not be valid in all cases. Indeed, sometimes the duplication unit is known to partially or completely exclude the promoter of the parental gene and hence the two daughter genes might substantially differ in their expression [41].

### Acquisition of new coexpressed partners by duplicate genes

We analyzed the same set of 698 independent duplicate genes and explored the change in the total number of partners of each duplicate gene pair (as indicated by *n*_1 _+ *n*_2 _- *n*_12_) with evolutionary time. Here we assumed that the average degree of a parental singleton gene before duplication is equal to the average degree of a singleton in the contemporary network. According to this assumption, immediately after gene duplication, the total number of partners of a duplicate gene pair is equal to that of a parental singleton gene, i.e. *n*_1 _+ *n*_2 _- *n*_12 _= *n*_s _(Figure 4B). Following duplication, as the two genes diverge in their expression profiles, their partners can be classified into three groups (Figure 4): (1) partners inherited from a parental singleton gene and still shared between the two genes in a pair (*shared ancestral partners*); (2) partners inherited from the parental singleton gene but present now only in one of the two duplicates (*unique ancestral partners*); and (3) new partners acquired independently by one of the duplicates (*unique acquired partners*). The present study does not allow us to differentiate between unique ancestral and unique acquired partners directly, but we can make indirect inferences about their relative numbers.

In our data set, shared partners constitute a small fraction among the partners of a duplicate gene pair: on average lower than 6.6% (see above). If, on average, following duplication, *n*_1 _+ *n*_2 _- *n*_12 _<*n*_s _(Figure 4C), this indicates loss of ancestral partners by duplicate gene pairs. If *n*_1 _+ *n*_2 _- *n*_12 _≈ *n*_s _(Figure 4D), this can be explained by the presence of a small fraction of shared partners and a large fraction of unique ancestral partners, i.e. all of the original partners of parental genes might still be retained by a duplicate pair (although some of the partners for a duplicate gene pair might be unique acquired partners, in this case such acquisition is compensated by a loss of ancestral partners). If, however, *n*_1 _+ *n*_2 _- *n*_12 _> *n*_s _(Figure 4E), an excess of an average number of partners for duplicate pairs over that for singletons can be explained by acquisition of new partners.

**Figure 6:**
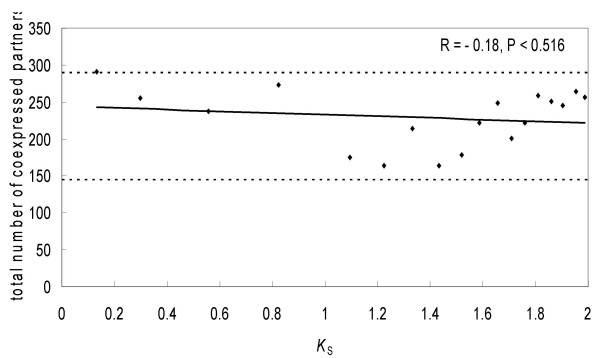
**The change in the total number of coexpressed partners with evolutionary time (measured by *K*_S_)**. Each point represents an average value for 40 duplicate gene pairs. The lower dashed line is the average number of partners for a singleton and the upper dashed line is twice the average number of partners for a singleton.

The average number of partners for duplicate gene pairs in our data set was significantly (~57%) greater than that for singleton genes (227.9 *vs*. 144.9; *t *= 10.57; *P *< 0.001, significance assessed by permutation test), suggesting acquisition of new partners by duplicate gene pairs. This suggests that on average more than one third of partners of a duplicate gene pair were acquired after duplication and not inherited from a parental singleton gene. Such gain of new partners was rapid: even at low *K*_S _(*K*_S _= 0.13 for the youngest 40 gene pairs), members of a duplicate gene pair were already expressed together with 291.4 genes (on average), while a singleton gene was expressed together with 144.9 genes (on average). Depending on *K*_S_, we observed some variation (and some insignificant decline) in the average total number of partners for a duplicate gene pair (Figure 6). Importantly, at any time point examined (0.13 <*K*_S _<2), *n*_1 _+ *n*_2 _- *n*_12 _was greater than *n*_*s*_.

### Asymmetric expression divergence of duplicate genes

**Figure 7:**
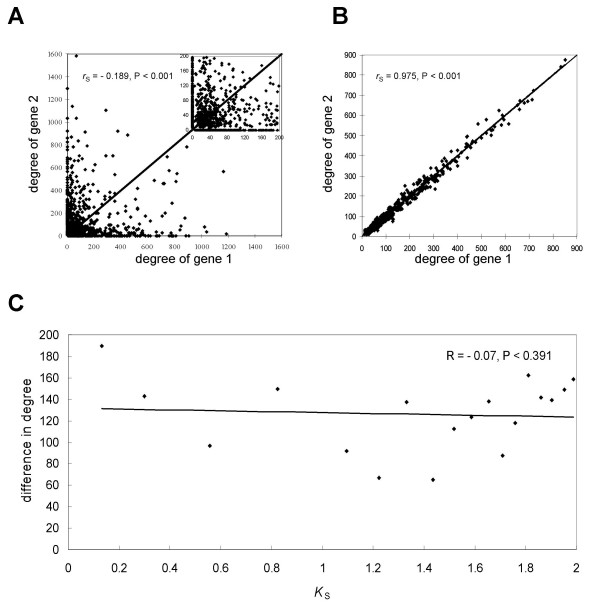
**Asymmetric divergence in gene expression**. (A) Plot of degree of one gene versus degree of another gene for 1,547 duplicate gene pairs with *K*_S _< 2 (inset shows pairs with both degrees below 200). (B) The same plot after numerical simulation of symmetric divergence with equal probability of loss and gain of coexpressed partners (*P *= 0.5). (C) The relationship between the difference in degree and time since duplication (measured by *K*_S_) for a pair of duplicate genes. Each point represents an average value for 40 duplicate gene pairs.

Two duplicate genes in a pair usually diverged asymmetrically in the network and this asymmetry was acquired quickly after duplication. For 1,547 independent duplicate gene pairs with *K*_S _< 2 (including pairs with only one copy in the network) we drew a scatter plot with the numbers of partners for two duplicate genes at the X and Y coordinates (Figure 7A). The assignment of a duplicate gene from each pair to either X or Y was random. Note that the plot predominantly reflected unique partners, since the proportion of shared partners was low (see above). Our simulations showed that if the divergence in gene expression were symmetric, we would expect a positive correlation between the numbers of partners for two duplicate genes in a pair (Figure 7B). However, in reality, we found a negative correlation (Figure 7A, Spearman's rank correlation coefficient *r*_s _= -0.19, *P *< 0.001), indicating that usually two duplicate genes had different numbers of partners. Interestingly, 849 out of 1,547 duplicate gene pairs were located on either the horizontal or vertical axis, suggesting that one gene had some partners in the network while the other one had none. Additionally, we observed that the difference in degree between duplicate genes in a pair was not related to *K*_S _(Figure 7C). Thus, the asymmetry in expression divergence was established early and was maintained throughout the evolutionary time examined.

### Robustness of the network

**Figure 8:**
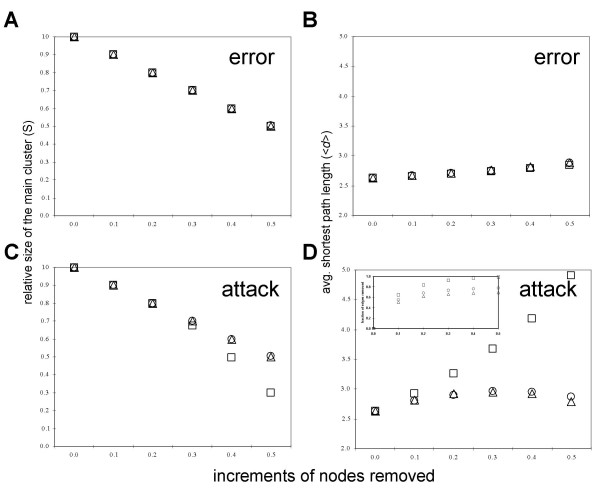
**The results of *in silico *perturbations of the network**. The effect of random removal of genes (error) on (A) the relative size of a giant cluster and (B) the average shortest path length. The effect of degree-based removal of genes (attack) on (C) the relative size of a giant cluster and (D) the average shortest path length (inset shows the fraction of edges removed). Singletons are marked by circles, duplicate genes by triangles, and all genes by squares.

To study the role of duplicate genes *vs*. singletons in the robustness of this coexpression network, we computationally perturbed the network by random removal of nodes (error) and degree-based removal of nodes (attack or the preferential removal of the most highly connected genes; [23]). Error and attack were performed separately on three categories of genes – singletons, duplicate genes, and all genes taken together. Thus, a total of six experiments were performed. In each experiment, we removed nodes in 10%, 20%, 30%, 40%, and 50% increments calculated from the total number of nodes in the network. The relative size *S *(the fraction of nodes in the giant connected cluster after node removal) and the average path length <*d> *of the largest connected cluster were measured at each increment (Figure 8). The decrease in *S *and increase in <*d> *indicate network breakdown.

Random removal of duplicate genes, singletons, or all genes had minimal effect on the network (Figures 8A and 8B). The size of the main cluster did not decrease beyond the reduction expected due to node removal and the average path length remained approximately constant, indicating that most unremoved nodes stayed connected. This error tolerance was expected due to the high connectivity and scale-free nature of the network [23] and to the similarity in degree distribution of the three categories of genes (Figure 1 and Additional file 4).

The network also appeared to be resilient to the degree-based node removal of either singletons or duplicate genes. Attack on singletons was expected to lead to a faster crash of the network than attack on duplicate genes because of the higher average degree of singletons (Table 1) and their higher proportion among hubs (Figure 3). At the 40% and 50% increments, the degree-based removal of duplicate genes indeed yielded a slightly smaller average path length (<*d>*_dup _= 2.92 *vs*. <*d>*_sin _= 2.96 at 40%; <*d>*_dup _= 2.79 *vs*. <*d>*_sin _= 2.86 at 50%; Figure 8D), thus providing marginal support for this expectation. However, contrary to the expectation, attack on singletons and attack on duplicate genes led to similarly minimal decreases in the relative sizes of the main cluster (Figure 8C).

In contrast with attack on either duplicate genes or singletons, attack on all genes severely damaged the network (Figures 8C and 8D). After 50% of all genes were removed by attack, the network broke down into many small clusters and as a result the relative size of the main cluster was only 0.30 as compared with 0.50 after error (Figures 8A and 8B). Additionally, this led to ~two-fold increase in the average shortest path length as compared with the effect of error (<d>_error _= 2.66 *vs*. <d>_attack _= 4.91, both after removal of 50% of nodes; Figures 8A and 8D).

Why did the network break down so rapidly after we attacked duplicate genes and singletons combined? We hypothesized that this could be due to the removal of a large number of hubs and edges. Indeed, although the same number of genes was removed in each experiment, more strong hubs were removed by attack on all genes (e.g., 20% of the strongest hubs for the 20% increment; Figure 8D) than on either duplicates or singletons separately (e.g., only ~10% of the strongest hubs in each case for the 20% increment; Figure 8D). Similarly, more edges were eliminated by attack on all genes (e.g., ~84% of edges for the 20% increment; inset in Figure 8D) than on either duplicates or singletons (~62% and ~68%, respectively, for the 20% increment; inset in Figure 8D).

## Conclusion

The analysis of duplicate genes in the human gene coexpression network allowed us to make the following conclusions. *First*, in agreement with analysis of yeast duplicate genes (e.g., [13,14,29,30]), our observations suggest that human duplicate genes quickly lose similarity in gene expression profiles. As a result, except for immediately after duplication, they cannot be considered redundant parts in the network. This might explain why the network was similarly tolerant to attack and error on either duplicate genes or singletons – rapidly after duplication, duplicate genes diverge in their expression profiles, reaching the level of similarity only slightly higher than that observed for random singletons. Since only 79 tissues were examined in our study, we cannot exclude a possibility that some additional links among duplicate genes could be revealed in other tissues and/or under different physiological conditions.

*Second*, the acquisition of new coexpressed partners was found to be rapid and to play a prominent role in the evolution of duplicate genes. This process might lead to attainment of new functions and operate as an engine creating diversity at the phenotypic level that is vital for adaptation. The importance of addition of new interactions was also pointed out in the yeast transcriptional regulation network, although there a net loss of interactions was observed [30]. The proportion of unique acquired partners is difficult to determine precisely in the present study, because we cannot directly distinguish between unique ancestral and unique acquired partners. For instance, as mentioned above, acquisition of new partners can be compensated by loss of ancestral partners and in this way will not be reflected in the total number of partners for a duplicate pair. In the future, experiments using expression information for an outgroup should allow one to differentiate between the two classes of unique partners and to provide more precise estimates of their numbers.

Although more evidence is necessary, asymmetry might represent a common scenario in the functional divergence of duplicate genes. Yeast, fruit fly, nematode, and human duplicate genes exhibit asymmetric divergence at the amino acid level [11,42,43]. Additionally, asymmetric divergence of protein-protein interactions was found for yeast duplicate genes [28].

In summary, this study provides an example of the use of duplicate genes to investigate the evolution of a gene coexpression network. By investigating the divergence of duplicate genes in the network, the speed and pattern of divergence within a network can be assessed. An alternative approach is to compare orthologous genes in the networks of different organisms [16]. Unlike the analysis of orthologs where divergence is determined by speciation time, utilization of paralogs provides an opportunity to inspect a range of divergence since duplications usually occur at various times in the evolution of a lineage (unless a duplication is due to a polyploidization event). A paralogous approach has an additional advantage of requiring sequence and expression information from just one genome. Utilization of paralogous and orthologous information is expected to provide complementary information and bring us closer to understanding network evolution.

## Methods

### Network construction

To map probe sets to genes, the exemplar and consensus sequences for U133A and GNF1H arrays [54] were used as queries to search against the longest transcripts of known and novel genes retrieved from Ensembl (NCBI build 34) using BLAST [44] with E = 10^-20^. The criteria of acceptable alignments were as described in [45]. Briefly, the alignment was accepted if (1) the identity was higher than 94% and the length was greater than either 99 bp or 90% of the length of the query, or (2) the identity was 100% and the length was greater than 49 bp. There were three cases: (1) a single probe set hit a single gene (9381 genes); (2) multiple probe sets hit a single gene (4961 genes and 13071 probe sets); (3) a single probe set hit multiple genes (18718 genes and 4377 probe sets). All genes and probe sets in case 1 were considered. In case 2, a probe set with the highest expression value (measured by average difference or AD) value was selected, similar to [15]. All genes and probe sets from case 3 were deleted due to potential cross-hybridization. As a result, we obtained a data set of 14,342 genes and probe sets with the one-to-one correspondence. Another data set (9,056 genes) represented a subset of the previous one from which the probe sets with suboptimal design (with _s, _x, _r, _i, _f, _g suffixes) were excluded. However, only the original data set is discussed henceforth since it contained a larger number of genes and the results obtained from the two data sets were similar. Following [37], genes with AD > 200 in a particular tissue were considered to be expressed in this tissue. The AD values were logarithmically transformed (with base equal to 2). Tissue-specific genes were defined as those expressed only in one tissue, and ubiquitously expressed genes were defined as those expressed in at least 78 out of 79 tissues.

A series of Perl and C programs (Additional file 5) were written to conduct the study. Two nodes were connected if they both were expressed in at least T common tissues with the Pearson correlation coefficient (calculated among these T common tissues) greater than R. We used an adjacency matrix *A *(Additional file 6) to store the topology of the network. The matrix stored binary symbols: "*a*_*ij *_= 1" indicated the existence of an edge between nodes *i *and *j *and "*a*_*ij *_= 0" indicated its absence. The matrix was symmetric with the diagonal equal to zero because of no inference of the direction of edges and no self-loops (simple graph). We focused on the genes located in the main (giant) cluster in which every gene was connected to every other gene by at least one path. Genes that formed small and isolated clusters were regarded as outside of the main network. The clustering coefficient, *c*, is defined as the ratio between the number of edges among nodes adjacent to *i *and the maximum possible, *k*_*i*_(*k*_*i*_-1)/2 [46]. The clustering coefficient approaches 1 if the neighbors of a node are connected to each other. The shortest path length was calculated according to the Floyd-Warshall's all pairs shortest paths algorithm [47].

Transitivity is a property of networks that are based on correlation coefficients of gene expression values [16]. If gene A is correlated in expression with gene B and gene B is correlated in expression with gene C, then gene A might be correlated with gene C. However, as mentioned by Jordan et al. [16], the level of such transitive correlation is unknown. In the network investigated in the present study, a link was defined by two parameters – the number of tissues in which the two genes are expressed and the correlation coefficient of expression values. This led to decreased transitivity of the network. Indeed, the average clustering coefficient (*c*) of the network is only 0.16 (under high transitivity *c *is expected to approach 1). This can be explained by the following example. Let gene A be expressed in 20 tissues with expression values {*e*^*A*^_1_, *e*^*A*^_2_, ..., *e*^*A*^_20_}, gene B be expressed in the first 10 of these 20 tissues with expression values {*e*^*B*^_1_, *e*^*B*^_2_, ..., *e*^*B*^_10_}, and gene C be expressed in the other 10 of these 20 tissues with expression values {*e*^*C*^_11_, *e*^*C*^_12_, ..., *e*^*C*^_20_}. If the correlation coefficient between {*e*^*A*^_1_, *e*^*A*^_2_, ..., *e*^*A*^_10_} and {*e*^*B*^_1_, *e*^*B*^_2_, ..., *e*^*B*^_10_} is higher than 0.7, then genes A and B form a link. Similarly, if the correlation coefficient between {*e*^*A*^_11_, *e*^*A*^_12_, ..., *e*^*A*^_20_} and {*e*^*C*^_11_, *e*^*C*^_12_, ..., *e*^*C*^_20_} is higher than 0.7, genes A and C form a link. However, in this example genes B and C do not form a link because they are expressed in different tissues. Thus, transitivity is not an inherent feature of all genes in the network.

The 79 tissues studied by Su et al. [37] include six tissues that overlap with several other tissues in the data set. Each of these six tissues represents a more inclusive set of cells (usually an organ, e.g., the whole brain) as compared with its parts also present in the data (e.g., parts of brain). When we generated a separate network without these six tissues, only approximately 88% of connections were the same between this and original networks. Moreover, 84% of connections stayed constant when we removed any six tissues at random (repeated 10 times). This indicates that each tissue possessed unique expression information and we did not exclude any of them from the study.

### Identification of duplicate genes

Duplicate genes among 22,291 protein sequences of known and novel genes in Ensembl (NCBI build 34) were identified according to Gu et al. [48]. Briefly, each protein was used as a query to search against all other proteins using FASTA [49] with E = 10. The alignments were retained if: (1) the alignment length (*L*) was over 80% of the longer sequence, and (2) the identity (*I*) was ≥ 0.3 if *L *was over 150 amino acids or *I *≥ 0.06 + 4.8 *L*^-0.32(1+exp(-*L*/1000)) ^if otherwise. We deleted proteins if they formed a hit due to the presence of a repetitive element of the same family. A single-linkage clustering algorithm was carried out to assemble duplicate genes into families. As a result, 11,512 duplicate genes were assigned to 2,865 families. The complete protein-coding gene sequences were re-aligned using CLUSTALW [50]. The synonymous and nonsynonymous substitution rates per site (*K*_S _and *K*_*A*_, respectively) were calculated using the *YN00 *module [51] of PAML [52] implemented in PERL. To identify the set of independent duplicate gene pairs we proceeded as follows. First, within each gene family we sorted gene pairs by *K*_S _in the ascending order and selected the pair with the lowest *K*_S _(pairs with *K*_S _< 0.05 were excluded). Next, within each gene family, we selected other independent pairs (with genes that have not yet been selected) sequentially with increasing *K*_S_. This resulted in 4,997 independent gene pairs (Additional file 7). Only independent duplicate gene pairs were considered for the analysis of divergence of duplicate genes, however, all duplicate genes were considered for the analysis of robustness and of the differences between duplicate genes and singletons.

### Permutation tests

The permutation test was used to assess the statistical significance of the difference in the network measures between duplicate genes *vs*. singletons. First, we removed original labels and randomly relabeled genes keeping the numbers of genes of the two categories consistent with the original data set. Second, we calculated the 2-sample *t*-statistic [53]. Third, we repeated the process 1000 times and built a null distribution of the *t*-statistic. Finally, we compared the observed *t *value (true labeling) with its null distribution to determine the *P *value.

### Asymmetry analysis

To simulate symmetric divergence in gene expression, we followed the method developed by Wagner [28]. Briefly, the number of lost partners was randomly generated from binomial distribution *B*(*n*_1 _+ *n*_2 _- 2*n*_12_, *P*). We tested three possible scenarios: divergence by loss of function (*P *= 0), divergence by gain of function (*P *= 1), and equal probability of loss and gain of function (*P *= 0.5). To test each of the three scenarios we proceeded as follows. The ancestral number of partners for each pair was approximated by the sum of the current number of shared partners (*n*_12_) and of a random number (*n*_*l*_) generated from the binomial distribution *B*(*n*_1 _+ *n*_2 _- 2*n*_12_, *P*). The number of gained partners (*n*_*g*_) after duplication was approximated by *n*_1 _+ *n*_2 _- 2*n*_12 _- *n*_*l*_. Within each duplicate gene pair, the lost connections were assessed by *n*_*l*1 _~ *B*(*n*_*l*_, 0.5) and *n*_*l*2 _= *n*_*l *_- *n*_*l*1 _for the first and second duplicate copies, respectively. The reconstructed number of coexpression partners from the symmetric divergence model were (*n*_12 _+ *n*_*l*_) - *n*_*l*1 _+ *n*_*g*1 _and (*n*_12 _+ *n*_*l*_) - (*n*_*l *_- *n*_*l*1_) + (*n*_*g *_- *n*_*g*1_) for the first and second gene copies, respectively.

### Robustness analysis of the network

The degree-based and random node removals were performed separately for duplicate genes, singletons, and duplicates and singletons combined (a total of six experiments). In each experiment, nodes were deleted in five increments (*f*): 10% (1290 genes), 20% (2580 genes), 30% (3770 genes), 40% (5158 genes) and 50% (6449 genes). The same number of nodes was removed in each experiment. For instance, by attacking 10% of duplicates, we removed 1290 (10% of 12897) duplicate genes with the highest number of connections. Similarly, by attacking 10% of all genes, we removed 1290 of the genes with the highest connections. Two quantities were used to assess the damage to the network: *S*, the fraction of nodes in the giant connected cluster after node removal (the relative size of the giant cluster), and <*d*>, the average shortest path length between any two nodes in this cluster. If nodes stay connected except for those that are removed (minimal damage), *S *= 1-*f*. For random removal of nodes from a scale-free network, *S *≈ 1-*f *and <*d*> stays approximately constant until a considerable fraction of nodes is removed [23]. However, degree-based removal leads to a fast decay of *S *and an increase in <*d*> until the network breaks down into small isolated clusters. Thus, the average shortest path length tends to first increase (the overall system is still functioning but it takes longer to travel from one node to another) and to decrease later, after a certain number of nodes is removed [23].

## Authors' contributions

WYC and KDM designed the study, WYC conducted the study, RA, IA, and AN contributed expertise necessary to conduct the study, WYC and KM wrote the paper.

## Supplementary Material

Additional File 1 The numerical description of the networks Click here for file

Additional File 2 **Degree distributions of networks generated from a combination of thresholds: *T *(tissue) and *R *(Pearson correlation coefficient)**. The degree distribution for the network with T ≥ 7 and *R *≥ 0.7 is shown in Figure 1 Click here for file

Additional File 3 The scatter plots between clustering coefficient *c *and node degree *k *for (A) all genes, (B) ubiquitously expressed genes, and (C) nonubiquitously expressed genes. Click here for file

Additional File 4 The degree distribution of the studied network (*T *≥ 7 and *R *≥ 0.7) for (A) duplicate genes and (B) singletons. Click here for file

Additional File 5 Scripts for the study Click here for file

Additional File 6 Network binary matrix. Click here for file

Additional File 7 The set of independent duplicate gene pairs. Click here for file
