# Supplementary material for: Rapid and asymmetric divergence of duplicate genes in the human gene coexpression network
Source: BMC Bioinformatics. 2006 Jan 27;7:46. doi: 10.1186/1471-2105-7-46 (PMC1403810; doi:10.1186/1471-2105-7-46)
Supplement: Additional File 1 — The numerical description of the networks [file 1471-2105-7-46-S1.pdf]

Additional file 1.
The numerical description of the networks

Networks T / R		Number of nodes in the giant cluster *(n)*	Average degree ($\langle k \rangle$)	Average shortest path length ($\langle d \rangle$)	Average clustering coefficient ($\langle c \rangle$)
5	0.5	13212	904.07	1.94	0.29
7	0.5	12897	807.95	1.99	0.31
9	0.5	12656	753.56	2.07	0.33
5	0.7	13212	184.96	2.27	0.14
7	0.7	12897	132.40	2.64	0.16
9	0.7	12653	110.15	2.93	0.18
5	0.9	13184	15.84	3.62	0.03
7	0.9	9865	5.88	5.26	0.05
9	0.9	4708	5.42	6.67	0.12

Each row represents a network that was generated for a particular pair of thresholds (T and R).
