# Supplementary material for: Rapid and asymmetric divergence of duplicate genes in the human gene coexpression network
Source: BMC Bioinformatics. 2006 Jan 27;7:46. doi: 10.1186/1471-2105-7-46 (PMC1403810; doi:10.1186/1471-2105-7-46)
Supplement: Additional File 2 — Degree distributions of networks generated from a combination of thresholds: T (tissue) and R (Pearson correlation coefficient). The degree distribution for the network with T ≥ 7 and R ≥ 0.7 is shown in Figure 1 [file 1471-2105-7-46-S2.pdf]

Additional file 2.

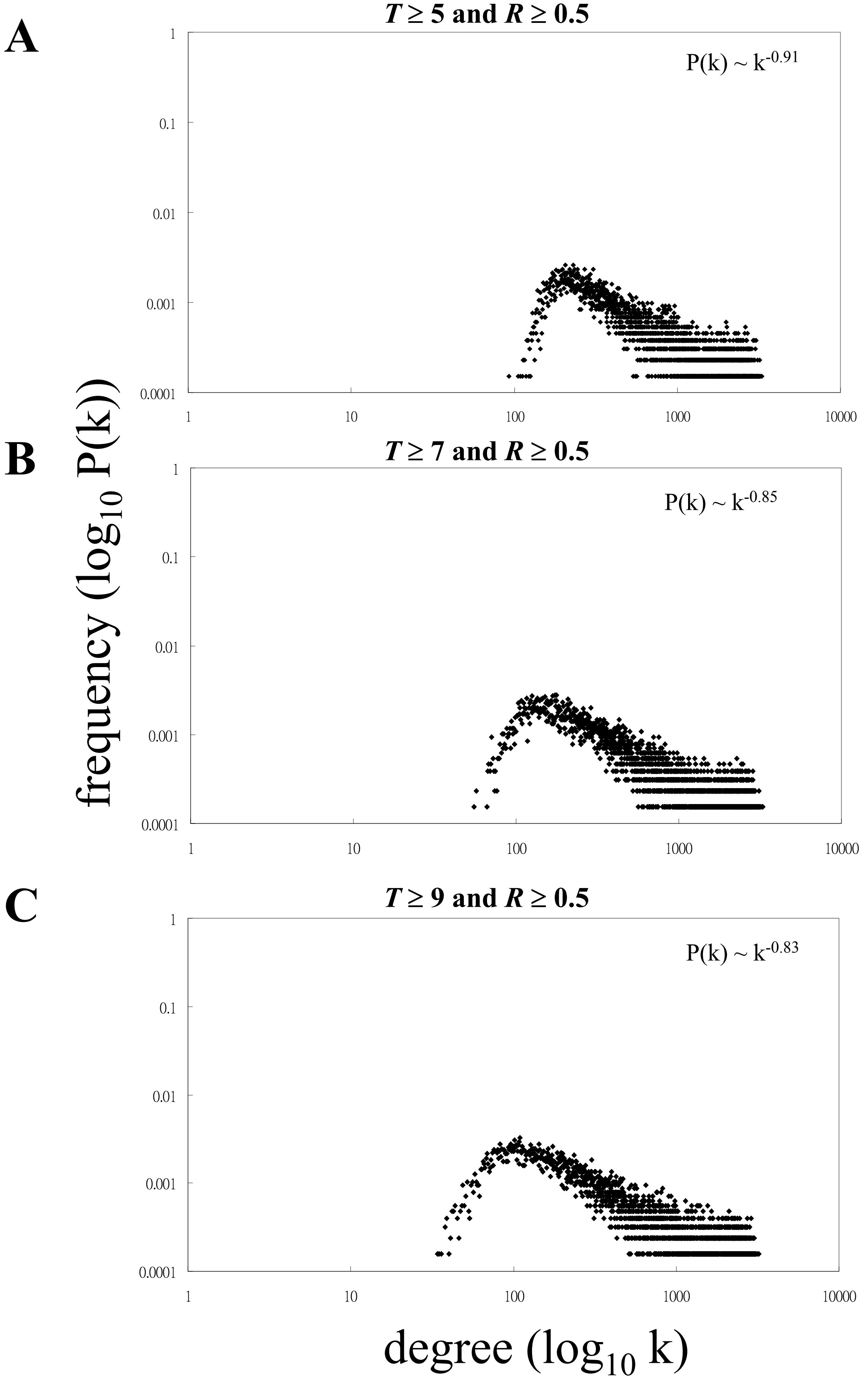

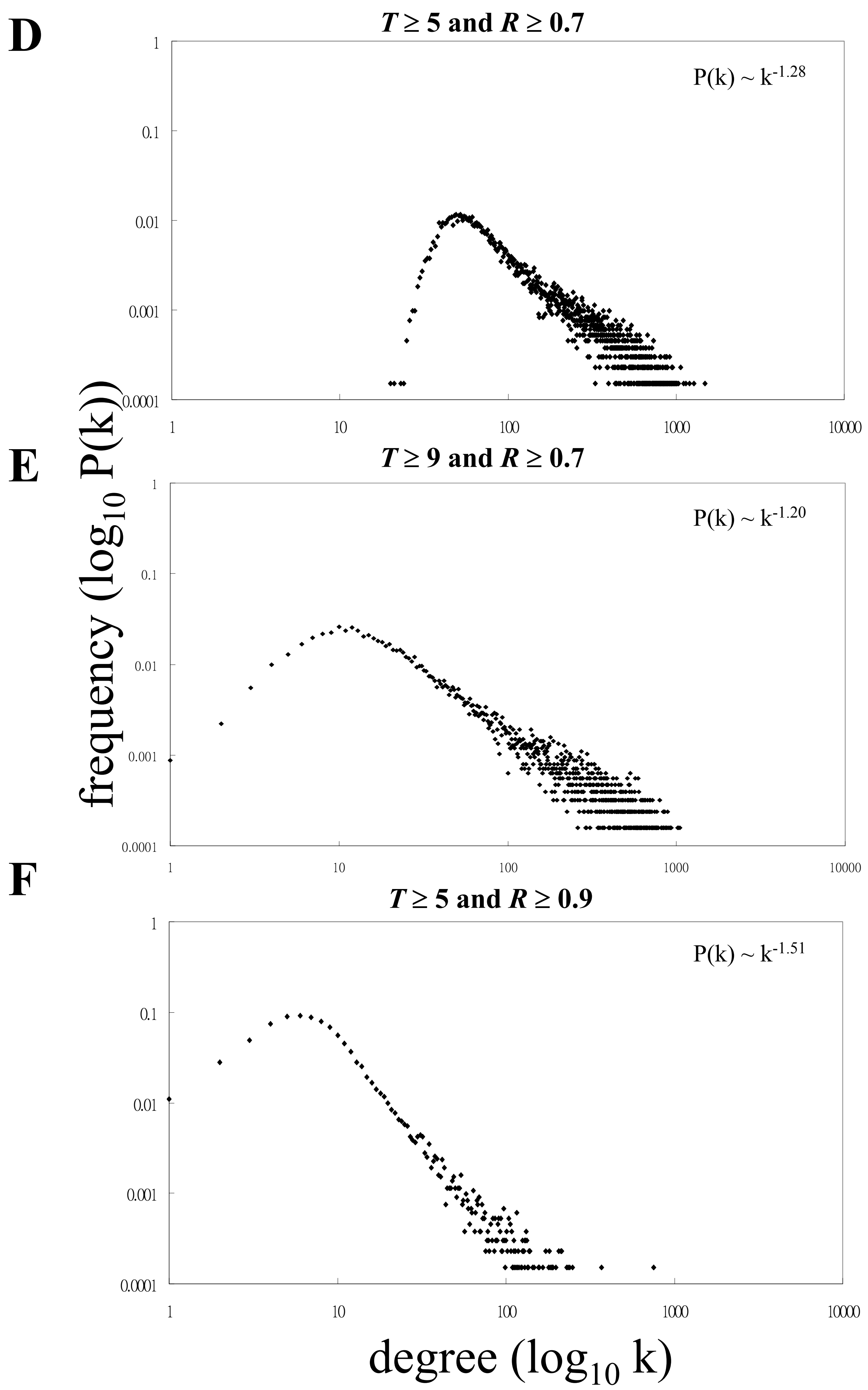
D
$T \geq 5$ and $R \geq 0.7$
$P(k) \sim k^{-1.28}$
E
$T \geq 9$ and $R \geq 0.7$
$P(k) \sim k^{-1.20}$
F
$T \geq 5$ and $R \geq 0.9$
$P(k) \sim k^{-1.51}$
1
0.1
0.01
0.001
0.0001
1
10
100
1000
10000
frequency ($\log_{10}$ P(k))
degree ($\log_{10}$ k)

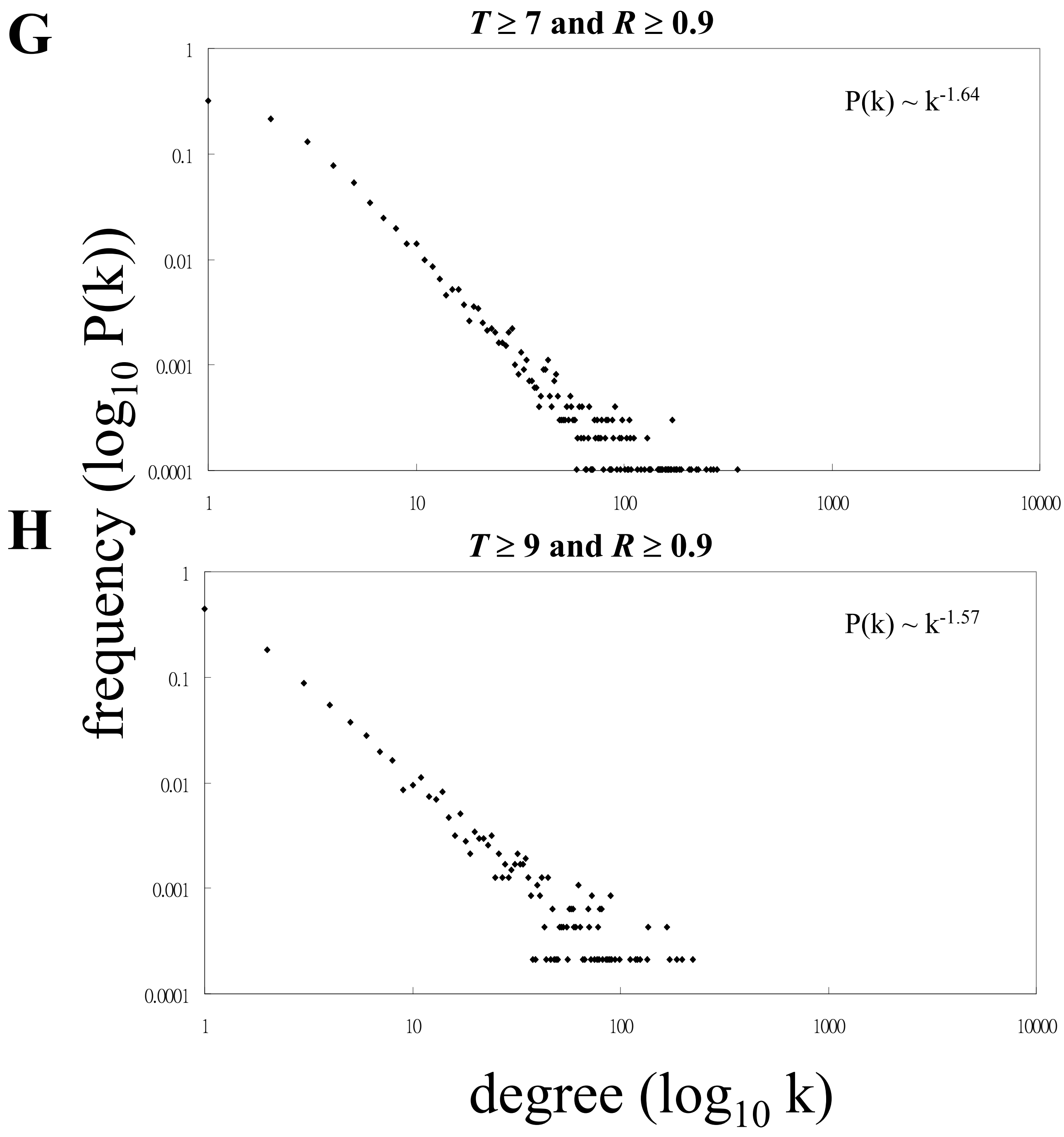
G
T ≥ 7 and R ≥ 0.9
P(k) ~ k-1.64
H
T ≥ 9 and R ≥ 0.9
P(k) ~ k-1.57
frequency (log10 P(k))
degree (log10 k)
1
0.1
0.01
0.001
0.0001
1
10
100
1000
10000
