# Supplementary figures and images for: Rapid and asymmetric divergence of duplicate genes in the human gene coexpression network
Source: BMC Bioinformatics. 2006 Jan 27;7:46. doi: 10.1186/1471-2105-7-46 (PMC1403810; doi:10.1186/1471-2105-7-46)

# Additional file 3.

A

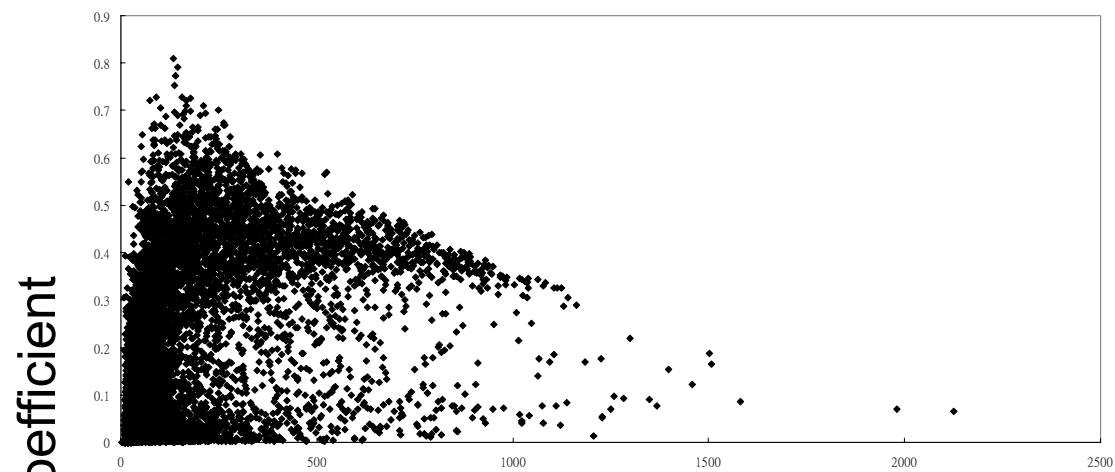

B

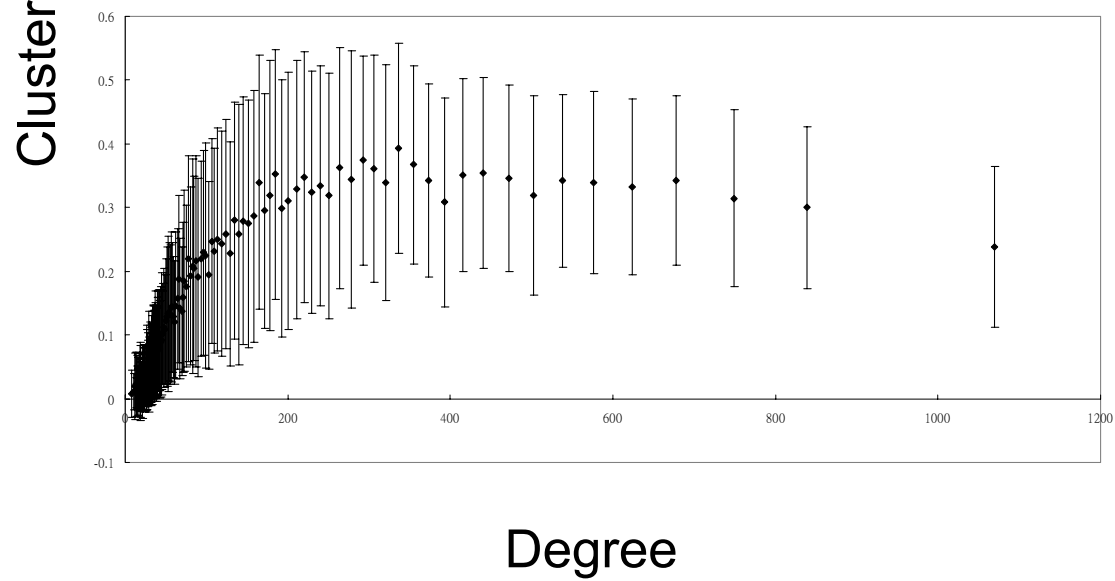

C

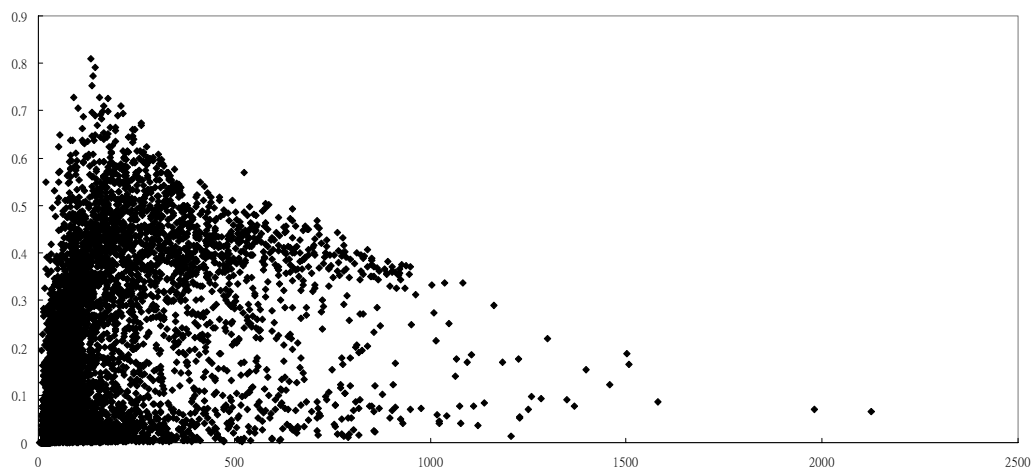

D

Clustering coefficient

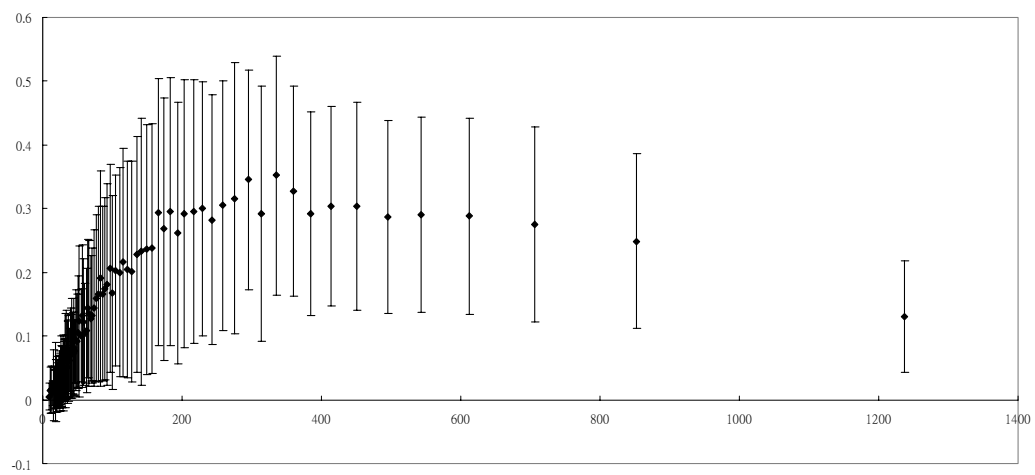

Degree

E

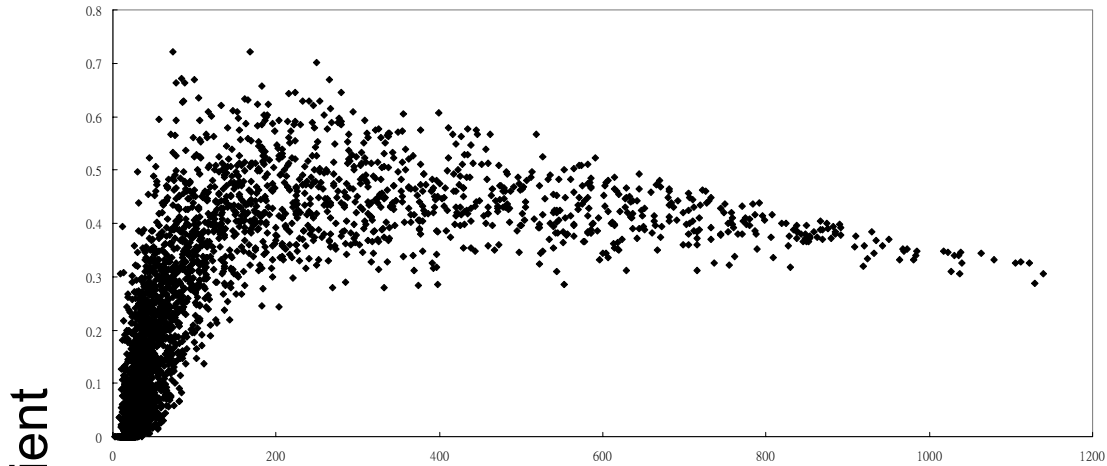

F

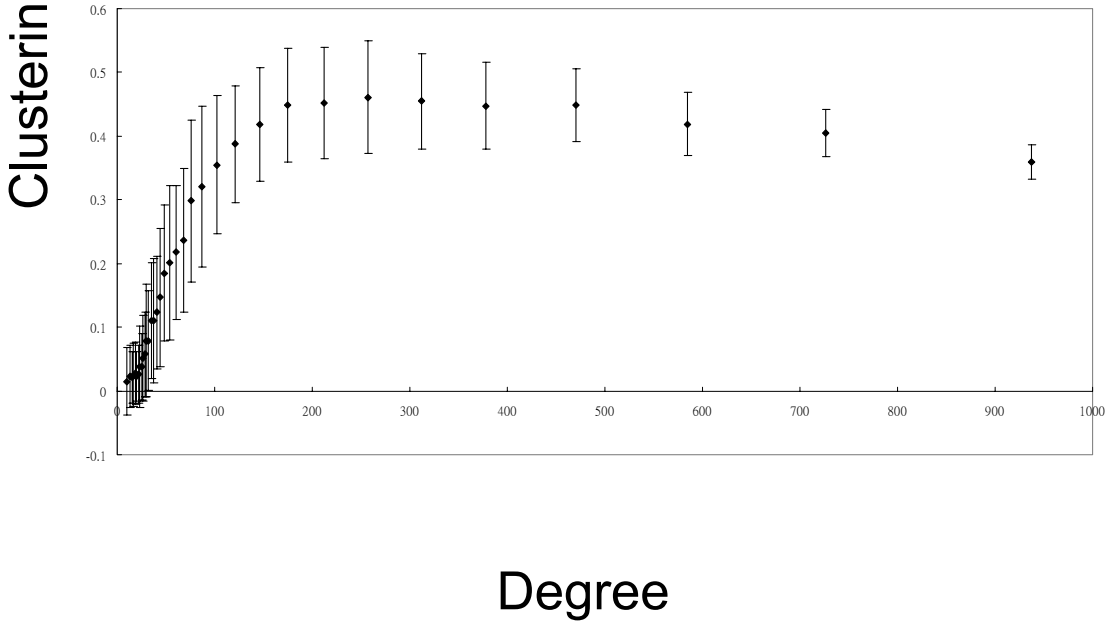

Supplement: Additional File 3 — The scatter plots between clustering coefficient c and node degree k for (A) all genes, (B) ubiquitously expressed genes, and (C) nonubiquitously expressed genes. [file 1471-2105-7-46-S3.pdf]

Additional file 4.

A

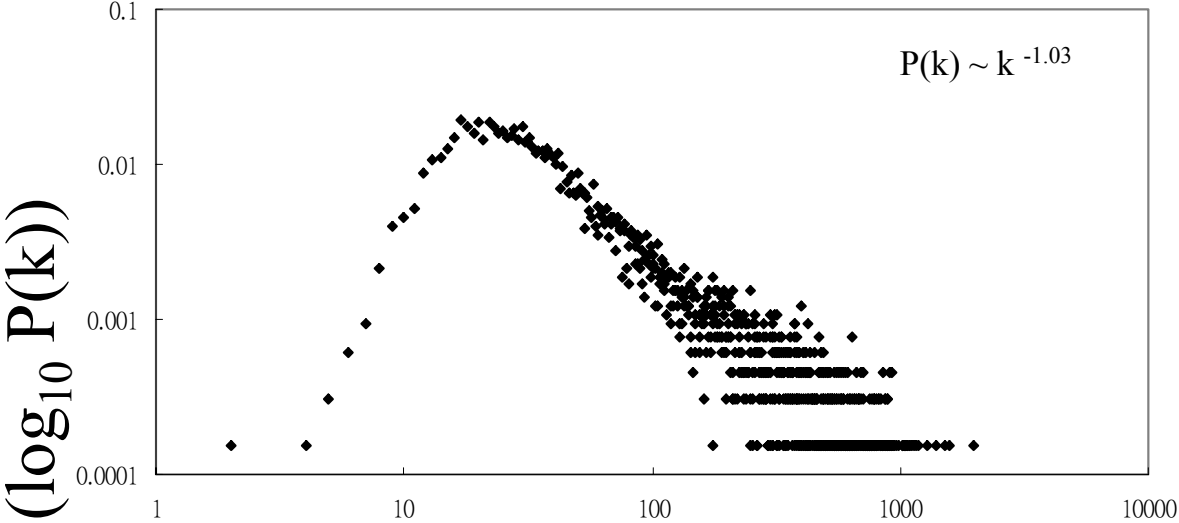

B

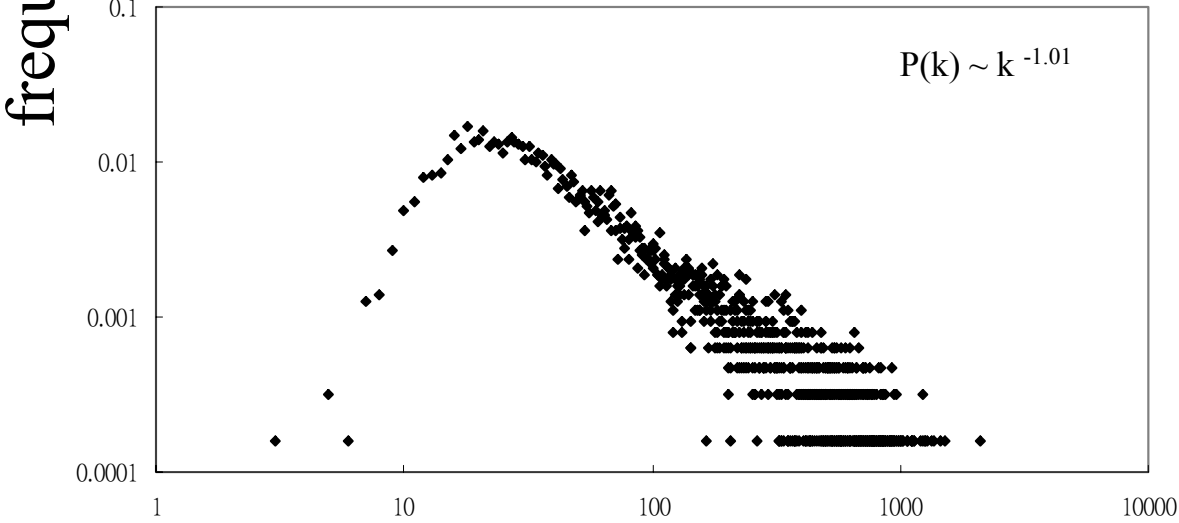

degree ( $\log_{10} k$ )

Supplement: Additional File 4 — The degree distribution of the studied network (T ≥ 7 and R ≥ 0.7) for (A) duplicate genes and (B) singletons. [file 1471-2105-7-46-S4.pdf]
